# Spatio-temporal low-level neural networks account for visual masking

**DOI:** 10.2478/v10053-008-0021-4

**Published:** 2008-07-15

**Authors:** Uri Polat, Anna Sterkin, Oren Yehezkel

**Affiliations:** Goldschleger Eye Research Institute, Tel-Aviv University, Sheba Medical Center, 52621 Tel-Hashomer, Israel

**Keywords:** temporal processing, visual masking, spatio-temporal interactions, temporal masking, visual evoked potentials, psychophysics

## Abstract

T emporal masking is a paradigm that is widely used to study visual information processing. When a mask is presented, typically within less than 100 msec before or after the target, the response to the target is reduced. The results of our psychophysical and visual evoked potential (VEP) experiments show that the masking effect critically depends on a combination of several factors: (1) the processing time of the target, (2) the order of presentation of the target and the mask, and (3) the spatial arrangement of the target and the mask. Thus, the masking effect depends on the spatial-temporal combination of these factors. Suppression was observed when the mask was positioned within a spatial range that was found to evoke inhibition, and when the temporal separation between the target and the mask was short. In contrast, lateral facilitation was observed when the mask was presented at a spatial separation that did not evoke inhibition from the target’s vicinity and with a temporal sequence that preceded the target, or when it was presented simultaneously with it, but not when the target preceded the mask. We propose that masking effects, either suppression or facilitation, reflect integration into the spatial and the temporal domains of the feedforward response to the target and the lateral inputs evoked by the mask (excitatory and/or inhibitory). Because the excitation evoked by the mask develops and propagates slowly from the mask’s location to the target’s location, it lags behind the response to the target. On the other hand, inhibition that is produced in the vicinity of the target evolves more rapidly and follows the onset and offset of the stimulus more closely. Thus, lateral excitation that overcomes the inhibition may facilitate the grouping of local elements into a global percept by increasing the survivability of the object and its accessibility for perceptual awareness.

## INTRODUCTION

Masking is a tool that is widely used to study information processing. When a mask is presented, typically less than 100 ms before or after the target, the target’s visibility is reduced – an effect that is usually inferred as suppression (Breitmeyer, 1984; Breitmeyer & Öğmen, 2000; Enns & Di Lollo, 2000; Francis, 2000). As the inter-stimulus interval (ISI) between the mask and the target increases, the masking effect is reduced; the time-window during which the target response is influenced by the mask can be interpreted as the time-window of interactions.

The masking effect is typically inferred from a comparison of the target’s visibility under two different conditions: the target alone and the target within the context of a mask. However, the neural representation of the same target under these two conditions may be different, and therefore, the masking should be probed by comparing between a local process (feedforward, target alone) and spatial integration that is context-dependent (feedforward and lateral interactions). The effects of context modulation, which may enable grouping of local elements into a global percept (Gestalt) were demonstrated in many psychophysical (Bonneh & Sagi, 1998; Herzog & Fahle, 2002; Kovacs, 1996; Polat, 1999; Polat & Sagi, 1993, 1994) and physiological studies (Bauer & Heinze, 2002; Chavane, Monier, Bringuier, Baudot, Borg-Graham, Lorenceau, & Fregnac, 2000; Kapadia, Ito, Gilbert, & Westheimer, 1995; Kapadia, Westheimer, & Gilbert, 2000; Li & Gilbert, 2002; Mandon & Kreiter, 2005; Mizobe, Polat, Pettet, & Kasamatsu, 2001; Polat & Norcia, 1996; Schmidt, Goebel, Lowel, & Singer, 1997; Sugita, 1999), for a review, see (Series, Lorenceau, & Fregnac, 2003). These studies clearly show that the neural representation of a target is modulated with regard to the surround stimuli. It is also apparent from these studies that the outcome of contextual modulation is complex; it is mostly suppressive but may also be facilitative in some spatial-temporal combinations. The nature (either facilitation or suppression) and the strength of the context effects are determined by several parameters such as proximity, similarity, contrast, and global configuration.

Traditionally, masking is treated separately in the spatial and temporal domains (Breitmeyer, 1984). In the temporal domain, when the mask precedes the target, it is termed forward masking (FM), whereas mask presentation following the disappearance of the target is termed backward masking (BM). Most of the temporal masking studies have focused on BM, less on FM, whereas simultaneous masking (SM) has been typically treated as a separate condition, most likely due to the lack of a temporal mismatch between the target and the mask.

In the spatial domain, the literature on masking distinguishes between pattern masking (mask and target presented at the same retinal location) and metacontrast (the mask location does not overlap with the target location, also termed lateral masking). This distinction is based on an implicit assumption that sharp boundaries that allow a visually apparent gap between the target and the mask indicate a distinct activation of different receptive fields. However, within the context of neuronal modeling, an important factor is the overlap between the receptive fields of the responding units, which may account for lateral interference regardless of whether the physical stimuli overlap or not. We will address this important issue next.

Our working hypothesis is that the masking effect critically depends on a combination of spatial and temporal stimuli attributes that can be summarized in a descriptive model with the following main factors: (1) the processing time of the target, (2) the presentation order of the target and the mask, and (3) the spatial arrangement of the target and the mask.

**1. Processing time.** An estimate of the persistence or the integration time of the target response taken from physiological experiments (Albrecht, 1995; Mizobe et al., 2001; Polat, Mizobe, Pettet, Kasamatsu, & Norcia, 1998) provides an upper limit of 200 ms. This estimate is consistent with psychophysical results showing that the integration time for contrast detection at threshold is 160-200 ms (Watson, Barlow, & Robson, 1983) and with results from our laboratory (Rosen, Belkin, & Polat, 2005). We assume that a mask presented beyond this time-window will fail to affect the response to the target.

**2. Interactions: excitation vs. inhibition.** The results of Polat and Sagi (2006) showed that temporal masking is affected by the order of presentation of the target and the mask as well as the spatial separation between them, which can be explained by the temporal and spatial properties of excitation and inhibition. 

*Dynamics*. Temporal masking can be accounted for by assuming different time courses for excitatory and inhibitory interactions. Whereas excitation develops slowly and is sustained, lagging behind the stimulus both in onset and offset, inhibition is rapid and transient, thus following the onset and offset of the stimulus more closely.

*Spatial architecture*. Several models of lateral interactions assume that excitatory and inhibitory connections form a neuronal network that determines the measured responses (Adini & Sagi, 2001; Adini, Sagi, & Tsodyks, 1997; Polat, 1999; Polat et al., 1998). It is assumed that each network unit receives three types of visual input: (1) direct thalamic-cortical input, (2) lateral input from other units within the network, and (3) top-down feedback. These inputs can be subdivided into excitatory and inhibitory types. The lateral excitation is organized along the filters’ optimal orientation, forming a collinear field (Polat & Norcia, 1998; Polat & Tyler, 1999), and is superimposed on a suppressive area surrounding the filters.

*Propagation time*. It has been suggested that the size of the receptive fields in V1 is estimated to be between 2 to 3λ (Mizobe et al., 2001; Polat, 1999; Polat & Norcia, 1996; Polat & Sagi, 1993; Watson et al., 1983; Zenger & Sagi, 1996). Thus, masking effects from target-to-mask separations of 2λ or less may be considered as integration (or summation) within the same receptive field (pattern masking), whereas separations of 3λ or more activate lateral interactions between different neurons responding to the target and the mask (lateral masking). Masking effects from outside the receptive field propagate to the target’s location through lateral connections, which are relatively slow compared with the direct input received by the receptive field. The estimated propagation speed of lateral excitation derived from psychophysical studies is about 3 degrees per sec (Cass & Spehar, 2005; Tanaka & Sagi, 1998), in agreement with the estimates from intracellular and optical imaging measurements (Bringuier, Chavane, Glaeser, & Fregnac, 1999; Malonek, Tootell, & Grinvald, 1994; Series et al., 2003). Therefore, facilitation is possible only if the propagation of the excitatory input from the mask to the target is not delayed by a period longer than the integration time of the feedforward input.

**3. Pattern vs. lateral masking**. Most of the masking studies used targets and masks that can be regarded as broadband stimuli in the spatial domain, and thus may be detected by receptive fields of different sizes. Therefore, it is likely that larger receptive fields respond both to the target and the mask. Thus, the masking effect may be related to interactions within the same receptive field, resulting in pattern masking. For example, in these studies it is impossible to differentiate between pattern and lateral masking, and the observed results may be confounded by both types of masking. Thus, an important factor in masking is the overlap between the receptive fields of the responding units, which may account for lateral interference, regardless of whether the physical stimuli overlap or not.

 In this study we also sought to find the neurophysio-logical correlates for the masking effect with the same stimuli that we used in the behavioral BM experiment and to compare our observations with previous findings in the literature. A particularly relevant EEG study by Jeffreys and Musselwhite (1986) investigated whether metacontrast-related inhibition or suppression is reflected in early components of the waveforms in visual evoked potentials (VEPs), namely the C1 and C2 components. Scalp distributions of C1 and C2 reflect the respective sites of origin in the striate and extrastriate visual cortex (Jeffreys, 1971; Jeffreys & Axford, 1972). No effect of metacontrast masking was found in C1 or C2 amplitudes; however, a clear U–shaped masking function in a separate psychophysical study was observed. An earlier EEG study (Schiller & Chorover, 1966) did not find evidence for metacontrast masking effects in early VEP components as well. Bridgeman’s reanalysis of Jeffreys & Musselwhite’s data (Jeffreys & Musselwhite, 1986) revealed a U–shaped modulation of the VEP amplitude of a later visual component in the VEP, around 250 ms, corresponding to the behavioral U–shaped masking function, which was thought to reflect visual masking due to recurrent processing (Bridgeman, 1988). A modulation around this latency has been found in single neuron activity in the cat and monkey striate cortex (Bridgeman, 1975, 1980). Interestingly, a recent MEG study compared meta- contrast masking with variable stimulus onset asynchrony using effective vs. pseudo mask (van Aalderen-smeets, Oostenveld, & Schwarzbach, 2006). In order to determine whether the perceptual effect on the target’s visibility is reflected in the corresponding component of the VEPs, around 250 ms, a control condition was introduced – a pseudo mask. In contrast to an effective mask, the pseudo mask did not share similar features but otherwise was similar to the effective mask (similar physical qualia, different shape). The pseudo mask did not produce behavioral masking. However, the lack of a distinction in the VEPs’ amplitudes, around 250 ms, between trials presenting effective vs. pseudo masks, led to the conclusion that this late visual component cannot be taken as evidence for effective backward metacontrast masking. On the other hand, a post-perceptual component, around 340 ms, located over the temporal-parietal cortex, clearly showed the effect of visibility. The latter finding was interpreted as a contribution of working memory-related processes to metacontrast. Results of this study challenge Bridgeman’s conclusion, suggesting that the observed U-shaped modulation of VEP amplitude may reflect temporal interactions between the target and the mask, unrelated to the target’s visibility. However, the spatial characteristics of the mask, such as its shape, the sharpness of its edges, and the possibility of a consequent overlap with the visual field of the target are of critical importance (see factor 3 of our descriptive model). That is, the visually apparent lack of pattern masking does not necessarily guarantee the lack of overlapping between the target and the mask within the same receptive field.

Using VEP, we measured the interactions between the target and the subsequent mask at different temporal separations. We used the spatial separation that produces metacontrast masking (i.e., the target and the mask activate separate receptive fields) under conditions that provide behavioral facilitation of target visibility.

## METHODS

### Psychophysics

#### Participants

Ten subjects with normal or corrected-to-normal vision in both eyes participated in the experiments. Five subjects participated in the integration time experiment and another 5 in the backward masking experiment.

#### Stimuli

**Figure 1.**
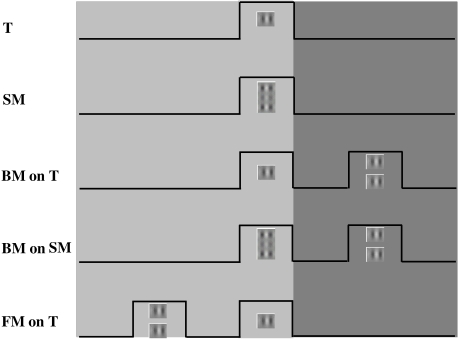
Example of stimuli used in this study. Three configurations of the target and masks were used in the temporal interaction experiments: simultaneous masking (SM), backward masking (BM), and forward masking (FM). The duration of presentation of the low-contrast target and the high-contrast mask was 60 ms in the psychophysical experiments and 50 ms in the VEP experiments. The masking effect was measured by comparing the responses under 5 conditions: (1) the target alone (T), (2) the target and mask presented simultaneously (simultaneous masking, SM), (3) the target followed by a mask (BM-on-T), (4) SM followed by a second mask (BM-on-SM), and (5) the target preceded by a mask (FM on T). The three levels of gray shading represent the three types of masking: forward, simultaneous, and backward.

**Figure 2.**
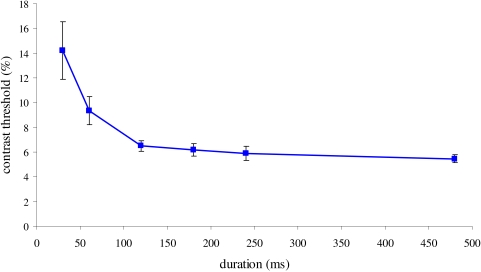
Integration time of a Gabor target at threshold. The contrast threshold (%, Y-axis) of the target detection for increasing durations of the target presentation (from 30 to 500 ms, X-axis) is shown (mean of 5 subjects ± SEM). The results show that the contrast threshold improves by more than a factor of two from the duration of 30 ms to 120 ms, followed by saturation.

The stimuli were localized gray-level gratings (Gabor patches) with a spatial frequency of 6 cycles per degree (cpd), modulated from a background luminance of 40 cd·m^-2^ (Fig. 1). Stimuli were presented binocularly on a Philips multiscan 107P color monitor, using a PC system. The effective size of the monitor screen was 24 × 32 cm, which, at a viewing distance of 150 cm, subtends a visual angle of 9.2 × 12.2 degrees. The subjects’ responses were recorded from a viewing distance of 150 cm, in a dark cubicle, wherein the only ambient light came from the display screen. The threshold of contrast detection was measured using a two alternative forced choice (2AFC) paradigm, in which the target had to be detected in one of two successive presentations, separated by an interval of 800 ms with a random jitter of 500 ms to avoid confounding the responses upon anticipation of the onset of the trial. A visible fixation circle in the center of the screen indicated the location of the target. Four visible crosses were presented at the corners of the monitor, at the same time with the target’s appearance, to avoid temporal uncertainty when presenting the target. The subjects activated the presentation of each pair of images (i.e., a single trial) at their own pace. Negative auditory feedback was provided. Contrast thresholds were measured utilizing a staircase method, which was shown to converge to 79% correct (Levitt, 1971). In this method, the target contrast is increased by 0.1 log units (26%), after an erroneous response, and is decreased by the same amount after three consecutive correct responses. About 40 trials were needed to estimate the threshold in each block. In addition, the threshold of contrast detection of the target presented alone, in a range of durations from 30 to 500 ms, was tested monocularly (Figure 2), whereas the rest of the parameters remained unchanged as in the rest of the experiments.

The masking paradigm included trials wherein the mask preceded the target (forward masking, FM), followed the target (backward masking, BM), or was presented simultaneously with the target (simultaneous masking, SM). The mask was composed of two Gabor patches, at a contrast of 40%, placed above and below the target, while the spatial distance between the target and each Gabor patch of the mask was constant in each experiment, either 2 or 3λ. The duration of the target and the mask presentation was 60 ms, while the ISIs between them were 0 ms (for SM), 60 or 80 ms (for BM), or -60 ms (for FM). Conditions under which a second mask appeared after the SM were included to explore the effects of backward masking on lateral interactions.

The mask with the shortest ISI constituted the first mask (M1), whereas the mask with a longer ISI constituted the second mask (M2). The masking effect was measured by comparing the detection thresholds under 5 conditions: (1) the target alone (T), (2) the target and mask presented simultaneously (simultaneous masking, SM), (3) the target followed by a mask (BM-on-T), (4) SM followed by a second mask (BM-on-SM), and (5) the target preceded by a mask (FM on T) (Fig. 1).

### VEP method

#### Participants

Five subjects with normal or corrected-to-normal vision in both eyes participated in the experiments.

#### Stimuli

The target, similar to the target stimulus used in the psychophysical experiments, was presented at 1 Hz for 50 ms at a contrast of 6% (at or very close to the detection threshold), with no change in the average background luminance. Backward masking, either on a target alone or on SM, was tested using stimuli similar to the masks used in the psychophysical experiments. The spatial distance between the target and the mask was 3λ. Each mask (either M1 or M2) was presented at the same spatial and temporal frequency, and for the same duration as the target. The mask was presented at ISIs of 0, 50, 150, or 250 ms (SOAs of 50, 100, 200, or 300 ms), following the target or the SM. The experimental conditions consisted of a target presented alone (T), SM, and all combinations of T or SM followed by masks at different ISIs (BM-on-T; BM-on-SM).

As in the psychophysical experiments, the mask with the shortest ISI constituted the first mask (M1), whereas the mask with a longer ISI constituted the second mask (M2). Each condition consisted of 10 trials (10 sec each), during which all the parameters were kept constant. Conditions were presented in random order. A small, 2-minute arc fixation point, located at the center of the screen, indicated the T location. Participants were instructed to maintain fixation and to avoid eye movements during the trials.

**VEP recording and signal processing**: The EEG was sampled at 432 Hz from a cruciform array of five electrodes centered at O_z_ and spaced by 3 cm. The recording channel with the highest statistical reliability (signal-to-noise ratio) was selected for group averages (the O_z_). For every condition, the average VEPs were computed over a 1000-ms period, for 10 identical runs, each composed of 10 stimulus presentations (trials, a total of 100 trials per condition). The mean of two periods of 1000 ms each, at the beginning and at the end of each run, was taken as the baseline for the run.

The amplitudes and the waveforms of the elicited responses for the various BM combinations were compared within time-windows defined according to the responses evoked by the T, M, and SM stimuli, at different delays relative to the beginning of the trial. The maximal amplitude of the first positive response peak was calculated in the corresponding time-window defined by the response to T or SM presented alone (P1 T or P1 SM). The maximal amplitude of the first positive response peak to the mask (i.e., the second positive peak response in the time courses under the BM conditions) was calculated in the time-window defined according to responses evoked by M presented alone at different delays relative to the beginning of the trial, corresponding to the different ISIs tested under BM conditions (P1 M1 or P1 M2). The maximal amplitude (in absolute terms, i.e., the maximal deflection from baseline) of the first negative response peak after P1 to T or SM was calculated in the time-window defined by the response to T or SM presented alone (N1 T or N1 SM). A prediction of the SM response was calculated as the sum of the time courses evoked by T and M, each presented alone at the onset of the trial (T+M). The correlation between the waveforms and/or the amplitude modulation was regarded as the BM effect. The correlation, unless a particular time-window was specified, was calculated for all the time courses; Peak amplitude comparisons between conditions were performed using the paired *t*-test.

## RESULTS

### Integration time

We first present data showing the integration time of the target (the threshold of contrast detection for a Gabor patch, 6 cpd) presented alone for a range of durations (Fig. 2). The results show that the contrast threshold improves by more than a factor of two from the duration of 30 ms to 120 ms, followed by saturation. This result is consistent with earlier results (Legge, 1978; Watson et al., 1983), indicating that efficient processing is performed during the first 120 ms of stimulus presentation, an observation that may pose an upper limit for efficient temporal masking.

### Effect of target-to-mask spatial separation

**Figure 3.**
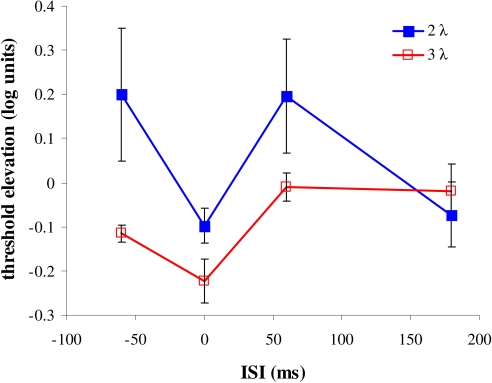
Asymmetric temporal interactions. The masking effect is shown (measured as the elevation of the threshold of the target detection) at different ISIs and target-to-mask separations. The X-axis denotes the temporal order of the mask presentation: negative values indicate forward masking (FM), zero indicates simultaneous masking (SM), and positive values indicate backward masking (BM). The Y-axis denotes the threshold elevation (positive values indicate suppression; negative values indicate facilitation). The blue line (closed squares) presents the results for target-to-mask separations of 2λ and the red line (open squares) for 3λ.

Our aim was to test the effect of spatial separation (i.e. the distance) on the masking effect. Two distances were tested: 2λ, which is assumed to have some overlapping with the target location, and 3λ, where no overlapping with the target location is assumed, as discussed in the Introduction and Discussion (Fig. 3). The masking effect was measured as the log of the target’s threshold, normalized to the threshold of the target presented alone (i.e., the threshold elevation). Thus, positive values indicate suppression, whereas negative values indicate facilitation. The results clearly show the effect of target-to-mask separation (2 and 3λ) and the asymmetry between the temporal conditions:

1) The effect of distance on temporal masking can be regarded as an effect from inside (2λ) and outside (3λ) the receptive field (see Introduction). Suppression is evident in BM and FM at 2λ, but not at 3λ. Facilitation is evident at 2λ only in SM, whereas at 3λ in FM and SM, but not in BM. For an ISI of 180 ms, however, no effect of temporal masking was found for any distance.

2) The results clearly show that SM, FM, and BM differ in the way they affect the target response at an ISI of 60 ms. FM produced facilitation at 3λ but resulted in suppression at 2λ. SM produced facilitation at both distances. BM produced suppression at 2λ, but had no effect at 3λ. Thus, the observed interaction between the effective integration time of the feedforward response and the delayed lateral response (due to a slow propagation time) seem to determine the perceptual masking effect.

### Difference between BM-on-T and BM-on-SM

It is possible that the asymmetric masking effect (FM vs. BM) observed above can be accounted for by differences between the temporal dynamics of the mask and the target responses and the interaction between them.

**Figure 4.**
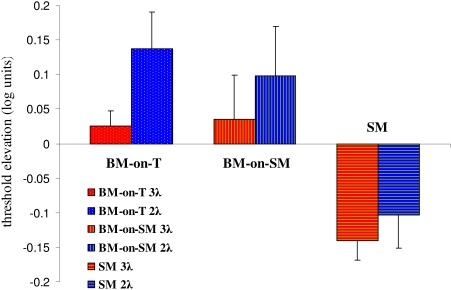
Comparison of BM-on-T, BM-on-SM and SM at 2 and 3λ. The elevation of the threshold of the target detection under the two BM conditions, as compared to SM, is shown. The Y-axis denotes the threshold elevation (positive values indicate suppression; negative values indicate facilitation). The results for BM-on-T (dots), BM-on-SM (vertical strips), and SM (horizontal strips) at 2 (blue) and 3λ (red) are presented (mean of 5 subjects ± SEM).

It was previously shown that when the mask in SM was presented continuously after the target disappeared (with no ISI), the effect of the facilitation expected in SM disappeared (Polat & Sagi, 2006). Here we investigated whether this temporal continuity of the mask presentation is necessary for abrogating the facilitatory effect of SM. BM-on-T was compared to BM-on-SM with both distances (2 and 3λ). The results, presented in Figure 4, clearly show that the facilitation at 3λ, which occurred during SM (*p* = .002, *t*-test), is not apparent when the same stimulus was followed by the second mask (BM-on-SM) (*p* = n.s., *t*-test). At a separation of 2λ, in SM there is significant facilitation (*p* = .03, *t*-test), whereas in BM-on-SM there is no facilitation (*p* = n.s., *t*-test). In BM-on-T, at 3λ no facilitation or suppression was observed (*p* = n.s., *t*-test), whereas at a separation of 2λ, there was suppression (*p* = .02, *t*-test). Thus, the appearance of a second mask at an ISI of 60 ms, after SM, interrupted the development of the expected facilitation. Similar results were observed for the 2λ separation.

### Effect of contrast in BM-on-SM

**Figure 5.**
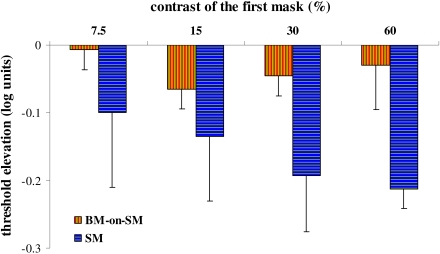
Effect of contrast of the first mask under BM-on-SM. The BM-on-SM condition was tested with different contrast levels of the first mask (from 7.5 to 60%, X-axis). The contrast of the second mask was kept constant, at 60% and the target’s contrast detection threshold in this experiment was 5%. The results are presented as threshold elevation (Y-axis, positive values indicate suppression; negative values indicate facilitation). The target-to-mask separation was 3λ. Orange bars – BM-on-SM condition; blue bars – SM condition.

It is still possible that the second mask under the BM-on-SM condition abrogates the facilitation observed in SM by inhibiting the response to the first mask by reducing its visibility. In other words, the effect might be regarded as pattern masking of the first mask by the second. If true, the perceived contrast of the first mask should be lower. It was shown earlier that even a low-contrast mask in SM still produces facilitation (Polat, 1999). Therefore, one would expect that reducing the perceived contrast of the first mask in BM-on-SM will still result in facilitation. We repeated the BM-on-SM experiment for different contrast levels (7.5-60%) of the first mask (Figure 5, orange bars). The contrast of the second mask was kept constant, at 60%. For comparison, the SM condition for the same mask contrasts was tested (Figure 5, blue bars). The results of the SM, presented in Figure 5, confirmed the earlier finding that facilitation is not dependent on the contrast of the first mask, and that this is valid between contrast levels of 7.5-60%, though the magnitude of the facilitation is slightly reduced for the lower contrast of the first mask. However, in BM-on-SM, the second mask abrogated the facilitation for all contrast levels (*p* < .0006, *t*-test), indicating that the effect of BM reduces the effective lateral interactions between the first mask and the target. Further support for this result comes from the VEP experiment, which is presented below.

### VEP data – temporal resolution of the target and mask responses

**Figure 6.**
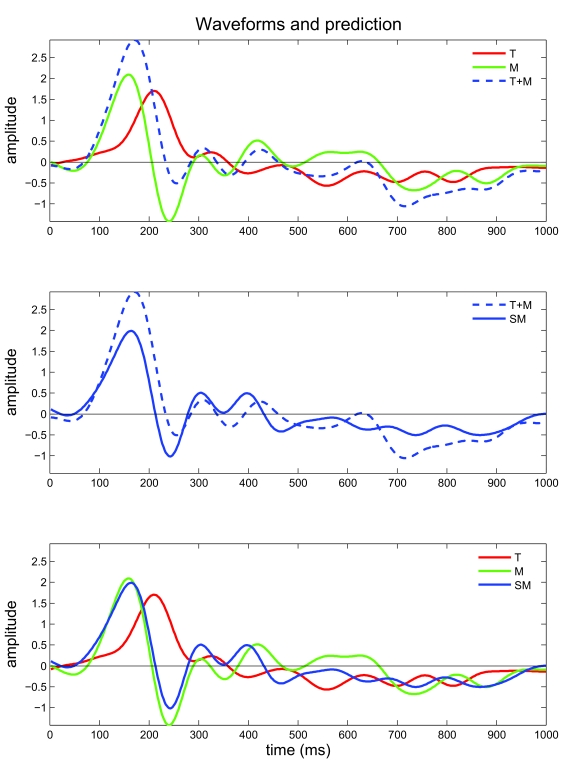
Evoked and predicted waveforms of visual evoked potentials. The average waveforms (time courses of 1000 ms, 5 subjects) of the visual evoked potentials (VEP) under three conditions are presented: the target presented alone (T, red line), the mask presented alone (M, green line), and the simultaneous masking (SM, blue line). The predicted time course for the SM (T+M, blue dashed line) was calculated as the linear sum of T and M. The time courses of T, F, and T+M are superimposed in the upper panel; T and T+M – in the middle panel; T, M, and SM – in the lower panel.

Figure 6 presents the time courses evoked by a low-contrast T presented alone, M presented alone, and SM, averaged for the 5 subjects, in comparison with the predicted SM response (T+M). The first positive amplitude (P1) of T is lower relative to P1 of M and SM. Moreover, P1 latency of T is delayed by 50 ms, compared with a P1 latency of F and SM (210 ms, 160 ms, 164 ms, T, M, and SM, respectively; averaged for 5 subjects) (Figure 6). Furthermore, a negative peak (N1) with a latency of 240 ms is evoked by M and SM, but not evoked by T.

The time course evoked by SM is significantly different from the predicted response (Figure 6), indicating a non-linear summation of the foveal and flanking components of SM. Moreover, although there is a very high correlation between M and SM (89.81%, *p* < .00001), there is a significant difference in the amplitude of the negative peak, N1, in the responses evoked under the two conditions (*p* = .035, *t*-test). Because the latency of the peak response to the T presented alone approaches the latency of the negative peak observed in M and SM, this difference between M and SM may reflect the contribution of the foveal low-contrast Gabor in SM.

**Figure 7.**
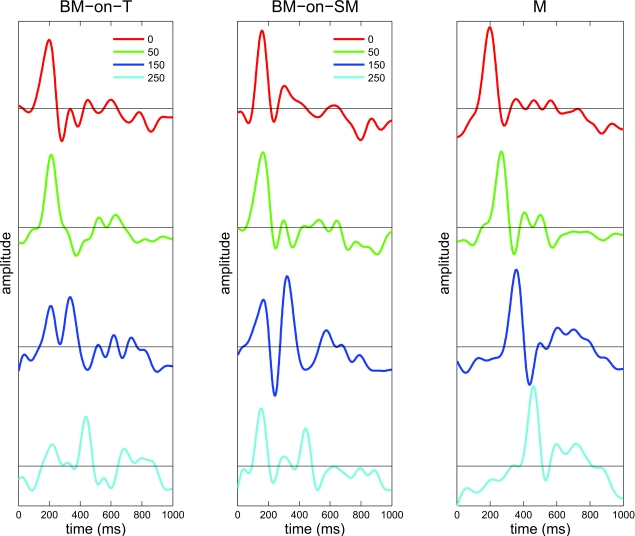
Waveforms of visual evoked potentials under BM-on-T and BM-on-SM. The average waveforms (time courses of 1000 ms, 5 subjects) of the visual evoked potentials (VEP) in a single subject under the two BM conditions, BM-on-T (left panel) and BM-on-SM (central panel) are shown. These waveforms are compared with the responses evoked by M presented alone at different delays relative to the beginning of the trial, corresponding to the different ISIs tested under BM conditions (right panel). The different ISIs tested under the BM conditions (0, 50, 150, or 250 ms) and the corresponding delays of M (50, 100, 200, or 300 ms) are coded with different colors.

**Figure 8.**
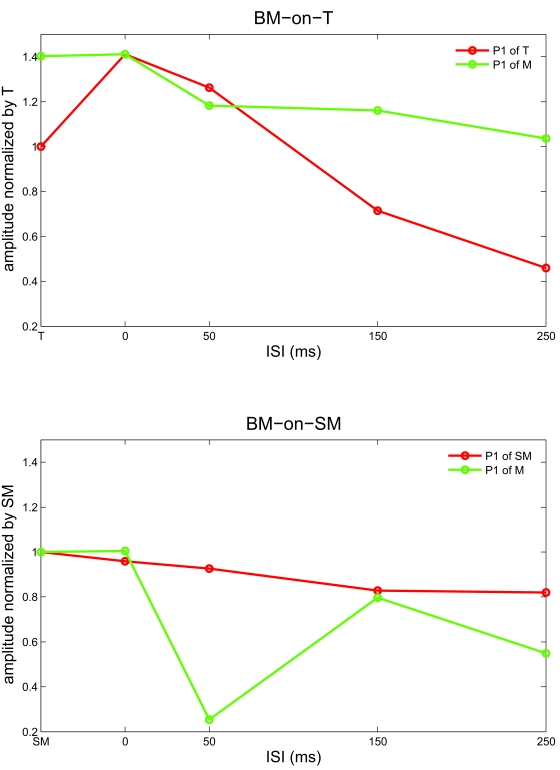
P1 amplitude under BM-on-T and BM-on-SM. The maximal amplitudes of the first positive response peak (P1) for the two successive stimuli presented under the two BM conditions at ISIs of 0, 50, 150, or 250 ms were calculated. The upper panel summarizes the results under the BM-on-T condition normalized by the P1 of T: P1 of T was calculated in the time-window defined by the response to T (red line); P1 of M was calculated in the time-window defined by the response to M, presented at different delays corresponding to the different ISIs (green line). The lower panel summarizes the results under the BM-on-SM condition normalized by the P1 of SM: P1 of SM was calculated in the time-window defined by the response to SM (red line); P1 of M was calculated in the time-window defined by the response to M, presented at different delays corresponding to the different ISIs (green line).

Figure 7 depicts the time course evoked under the two BM conditions, BM-on-T and BM-on-SM, at different ISIs, in comparison with the responses evoked by M presented at different delays, corresponding to the different ISIs tested under the BM conditions. Figure 8 summarizes the P1 values under the two BM conditions, both for the target and mask stimuli.

Under the BM-on-T condition, at an ISI of 0 ms (SOA = 50), the latency of the P1 of T (210 ms) coincides with the latency of the P1 of M (210 ms, i.e., 160 ms plus 50 ms of delay in onset). The waveform is highly similar to the SM condition (*p* = n.s., *t*-test; correlation of 91.5% with SM, first mask P1 time-window, maximal cross-correlation of 99.39%, achieved at a shift of 37 ms). Thus, BM with ISI = 0 results in a “fused” response pattern, i.e., as if the T and the M were presented simultaneously. Therefore, it is impossible to decompose the signal into independent responses to the T and M stimuli. At an ISI of 50 ms, the responses to T and M are not yet separated; however, starting from an ISI of 150 ms, the two response peaks (P1 T and P1 M) can be clearly separated. That is, there is a high similarity between the first positive peak with the P1 of T when presented alone (*p* = n.s., correlation of 88.24%, *p* < .00001, P1 of T time-window) and a second positive peak with the P1 of M when presented alone at the corresponding delay (*p* = n.s.; correlation of 86.45%, *p* < .00001, P1 of M time-window). At all ISIs except an ISI of 50 ms, the amplitude of P1 of T did not differ from the P1 amplitude of the target presented alone, whereas the amplitude of P1 of M remained similar to P1 of M when presented alone in all ISIs (Fig. 8).

Under the BM-on-SM condition, at an ISI of 0 ms, the second mask (M2), in fact, is a direct continuation of the first mask (i.e., a mask duration of 100 ms). The evoked response is similar to SM when presented alone (*p* = n.s., *t*-test; correlation of 86.7%, *p* < .00001), indicating that the additional 50 ms of mask duration do not affect the response. However, already at an ISI of 50 ms, the latencies of the P1 of SM (164 ms) and the mask are clearly separated (the latency of P1 of M is 260 ms). P1 of SM is highly correlated and has an amplitude similar to the time course of SM when presented alone (*p* = n.s., *t*-test; correlation of 99.46%, *p* < .00001, first mask P1 time-window), but P1 of M is significantly suppressed (*p* = .0277, *t*-test; no significant correlation with M when presented alone at the corresponding delay, *p* < .00001, second mask P1 time-window). However, for an ISI of 150 ms and longer, P1 of M almost “recovers” (*p* = n.s., *t*-test; correlation of 70.77% with M when presented alone at the corresponding delay, *p* < .00001, second mask P1 time-window). At an ISI of 250 ms, there is a high correlation between the first positive peak with the P1 of SM when presented alone (98.46%) and between the second positive peak with the P1 of M when presented alone with the corresponding delay (76.42%).

Regarding the N1 peak (a delay of 240 ms), a significant effect (i.e., a reduction of amplitude in absolute terms) under the BM-on-SM condition was observed only at an ISI of 50 ms (*p* = .0023, *t*-test), whereas under the BM-on-T condition there is a trend (although not significant) for a more negative N1 at an ISI of 0 ms.

## DISCUSSION

In this study our working hypothesis was that masking effects, either suppression or facilitation, reflect integration into the spatial and the temporal domains of the feedforward response to the target and the lateral inputs evoked by the mask (excitatory and/or inhibitory). It was found that when masking of a single target was explored, the expected suppression effect was observed for both FM and BM, but only with a spatial separation of 2λ (i.e., interactions within the same receptive field). However, facilitation was observed at 3λ (i.e., interactions between different receptive fields), with FM and SM, but not with BM. This complex pattern of results can be explained by two effects: (1) asymmetry between the processing of the target and the mask: the response to the mask is delayed behind the response to the target, due to the extra time required for the lateral propagation of the response from the mask to the target’s location. The lag between the responses to the mask and the target increases with increasing distance. Thus, the interaction between the two responses is possible only if the lateral propagation reaches the target’s location, within a limited time-window (efficient processing time). (2) Asymmetry between inhibition and excitation: whereas excitation develops slowly and is sustained, lagging behind the stimulus both at the onset and offset, inhibition is rapid and transient, following the onset and offset of the stimulus more precisely.

When backward masking was applied to a low-contrast target in the context of two higher-contrast masks (BM-on-SM condition), the results showed that BM affected the lateral facilitation induced by the first mask on the target and not the detection of the target per se. Therefore, we suggest that BM-on-SM abolishes the facilitation evoked by SM on the target. An alternative interpretation, according to which the second mask suppresses the response to the first mask, is ruled out both by the results of the psychophysical experiments (Figures 4, 5) and by the VEP results (Figure 8). Moreover, the VEP results show the opposite: the response to the second mask decreased, whereas the response to the first remained unchanged. Thus, the VEP, in concert with the behavioral findings, rules out the possibility of a pattern masking effect of the second mask on the first mask.

### The possible neuronal mechanism underlying masking

 What is the possible neuronal mechanism underlying the observed masking effects? Polat and Sagi (2006) suggested that both facilitation and masking reflect excitatory and inhibitory interactions within neuronal networks in response to Gabor stimuli (Adini et al., 1997; Hirsch & Gilbert, 1991; Polat et al., 1998). The presentation of a mask initiates both excitatory and inhibitory processes. However, whereas excitation develops slowly and thus lags behind the stimulus, inhibition is rapid and follows the onset and offset of the stimulus more closely. Thus, when the first mask is turned off, the inhibition decays rapidly, whereas the sustained excitation persists, resulting in lateral facilitation of the target. This suggestion is supported by the relatively slow time scale that characterizes lateral interactions (Bringuier et al., 1999; Malonek et al., 1994; Series et al., 2003) and strong, transient (Borg-Graham, Monier, & Fregnac, 1998) and fast inhibition (Bair, Cavanaugh, & Movshon, 2003). 

In the present study we highlight the importance of the temporal matching between feedforward input and lateral propagation, by monitoring their delays using VEP measurements. The response delay decreases with increasing target contrast by up to 100 ms (our unpublished data), which is consistent with data revealed from single unit recordings. Here we show that the delay of the peak response to the target presented alone was 210 ms (on average), whereas the corresponding delay of the mask response was 160 ms, indicating that the feedforward signal of the mask precedes the signal of the target (low contrast) by 50 ms. Because the speed of lateral propagation of the mask response is slow, it reaches the target’s location with a delay of an additional 50 to 100 ms (Polat & Sagi, 2006). Thus, the resulting delay of the lateral masking effect is 210 to 260 ms. As shown in Figure 2, an efficient integration time of the target (at the threshold of contrast detection) is about 100 ms. Therefore, the time-window for any efficient interactions with the target processing is from 210 to

310 ms after the onset of the target. Thus, any modulation of the response to the target by the mask may occur only if the responses to the target and the mask are temporally matched within this efficient processing time-window. Thus, in BM, when the mask that is presented at SOAs of 50 ms after the target (ISI of 0 ms), the mask response would propagate to the target location with a resulting delay of 260 to 310 ms, which is still within the efficient processing time-window, enabling the two signals to interact and produce a masking effect. In agreement with the above calculation, our results (Figure 7) show a waveform under the BM-on-T condition with an ISI of 0 ms, which is highly similar to the waveform evoked by SM. Note that the delay of the P1 response peak under this condition is exactly 210 ms. However, if the mask is presented with a long enough temporal separation (ISI of 150), the resulting delay of the mask response propagation to the target location is estimated at 410 to 460 ms, which is beyond the upper limit of an efficient processing time-window, resulting in no masking effect. And again, in agreement with the above calculation, our results show that the response to target under the BM-on-T condition at an ISI of 150 ms is similar in terms of amplitude and waveform to the target presented alone, indicating no masking effect.

In FM, when the mask is presented 50 ms before the target, the feedforward response to the target would be delayed by about 100 ms relative to the mask response. However, the lateral propagation of the mask response (with a delay of about 50 to 100 ms,

i.e., within the efficient processing time-window of the target) would modulate the feedforward processing of the target, resulting in a masking effect. In SM, the feedforward delay of the target (210 ms) is temporally matched with the resulting delay of the mask response (i.e., the sum of the feedforward delay of 160 ms and the lateral propagation delay of 50 to 100, which is 210 to 260 ms). Thus, the network response is biased towards excitation, resulting in facilitation of the response to the target.

### The inhibition-excitation account and its relationship to inside-outside the receptive field

A BM effect (suppression) on the target was observed for a target-to-mask separation of 2λ, but not of 3λ. The lateral masking effect is composed of inhibition and excitation. As previously mentioned, the inhibitory response is rapid and transient. As discussed above, in BM with ISIs of 50 to 100 ms, the rapidly developing inhibition coincides with the target response, which would result in a suppressive effect, but the relatively delayed excitation abrogates the inhibition. However, when the mask is positioned at a distance of 2λ (i.e., overlapping with the receptive field of the target), the dominant effect would be inhibitory. The strong inhibitory response is composed of the lateral component as well as the local one (from the vicinity of the receptive field of the target). The lateral propagation of the excitation produced by the mask towards the target representation is relatively fast, since the spatial separation of 2λ is relatively short. Therefore, the excitation is temporally matched with the stronger transient inhibition from within the receptive field of the target. Thus, the lateral excitation and the local inhibition interact within the integration time of the target. This explanation is consistent with the physiological study, showing that the main effect of temporal masking is evident only when the mask is positioned within a distance that overlaps with the receptive field (Macknik & Livingstone, 1998). When the separation between the mask and the target was increased, the masking effect disappeared, in agreement with earlier studies (Breitmeyer, 1984).

Usually the distinction between pattern and lateral masking is based on an implicit assumption that the sharp boundaries that allow a visually apparent gap between the target and mask are indicative of a distinct activation of the center and surround. However, within the context of neuronal modeling, an important factor is the overlap between the receptive fields of the units responding to the target and mask, which may account for lateral interference regardless of whether the stimuli overlap or not. Physiological studies that showed clear effects of surround modulations on the classical receptive field (Kapadia et al., 1995; Mizobe et al., 2001; Polat et al., 1998), positioned the mask at a distance that, when presented alone, evoked no response from the target location. Thus, the masking effect may possibly be confounded by mixed responses from the target’s location as well as from the mask’s location. Therefore, we propose that pattern and lateral masking may be inseparable in some of the temporal masking studies, especially for stimuli presented in periphery.

### Is the VEP just a linear summation of the target and mask responses?

It has been suggested that changes in the early components of the VEP signals reflect linear summations of the waveforms but not the real perceptual effect (van Aalderen-Smeets et al., 2006). However, our VEP results show that the measured signals are very different from the prediction of a linear summation of the target and mask waveforms, whereas there is an interaction between the target and the mask (i.e., for ISIs of up to 50 ms). However, for ISIs longer than 150 ms, the mask and the target responses are independent (and thus equal to the prediction of a linear summation). Consequently, at such ISIs no masking effect is evident. Thus, the evoked potentials seem to mirror the reported perceived masking effect. Moreover, the negative peak response, N1, was found to be markedly reduced (in absolute terms) under the BM-on-SM condition at an ISI of 50 ms, as opposed to (van Aalderen-Smeets et al., 2006), who did not observe any effect of BM at this delay. It is possible that the “pseudo” mask, although having different features from the effective mask, may still have interfered with the receptive field of the target, in a way similar to that of the effective mask, thus producing an undistinguishable pattern of interference with the target processing in the physiological results. The psychophysical findings for the two types of masks, although differential, are influenced by both the perceptual and the cognitive (i.e., post-perceptual) components of the behavioral response.

Our results suggest that the masking effects, either suppression or facilitation, reflect integration in the spatial and temporal domains of the feedforward response to the target and the lateral inputs evoked by the mask (excitatory and/or inhibitory). The excitation evoked by the mask is relatively delayed to the target stimulus, because it develops and propagates slowly from the mask’s location to the target’s location. The inhibition produced in the vicinity of the target, however, evolves more rapidly, and therefore follows the onset and offset of the stimulus more closely. It is also possible that the temporal properties of the responses in our study can be accounted for by the dual-channel model, which assumes effects of transient inhibition on sustained excitation (Breitmeyer, 1984). However, our model differs from the dual-channel model in assuming that both inhibition and excitation remain active as long as the stimulus is present. Moreover, our model and results disagree with the model of object-substitution masking (Enns & Di Lollo, 2000) in showing that rather than being unaffected, as expected by the model, the response to the mask is reduced.

To conclude, the interplay between the sustained lateral excitation and the transient inhibition may facilitate the grouping of local elements into a global percept by increasing the survivability of the object and its accessibility for perceptual awareness.
